# High Blood Pressure in Children and Adolescents: Current Perspectives and Strategies to Improve Future Kidney and Cardiovascular Health

**DOI:** 10.1016/j.ekir.2022.02.018

**Published:** 2022-03-01

**Authors:** Cal H. Robinson, Rahul Chanchlani

**Affiliations:** 1 Division of Pediatric Nephrology, Department of Pediatrics, The Hospital for Sick Children, Toronto, Ontario, Canada; 2 ICES (Formerly known as Institute of Clinical Evaluative Sciences), Ontario, Canada; 3 Department of Health Research Methods, Evidence and Impact, McMaster University, Hamilton, Ontario, Canada; 4 Division of Pediatric Nephrology, Department of Pediatrics, McMaster University, Hamilton, Ontario, Canada

**Keywords:** blood pressure, cardiovascular health, children, hypertension, kidney disease, pediatric

## Abstract

Hypertension is one of the most common causes of preventable death worldwide. The prevalence of pediatric hypertension has increased significantly in recent decades. The cause of this is likely multifactorial, related to increasing childhood obesity, high dietary sodium intake, sedentary lifestyles, perinatal factors, familial aggregation, socioeconomic factors, and ethnic blood pressure (BP) differences. Pediatric hypertension represents a major public health threat. Uncontrolled pediatric hypertension is associated with subclinical cardiovascular disease and adult-onset hypertension. In children with chronic kidney disease (CKD), hypertension is also a strong risk factor for progression to kidney failure. Despite these risks, current rates of pediatric BP screening, hypertension detection, treatment, and control remain suboptimal. Contributing to these shortcomings are the challenges of accurately measuring pediatric BP, limited access to validated pediatric equipment and hypertension specialists, complex interpretation of pediatric BP measurements, problematic normative BP data, and conflicting society guidelines for pediatric hypertension. To date, limited pediatric hypertension research has been conducted to help address these challenges. However, there are several promising signs in the field of pediatric hypertension. There is greater attention being drawn on the cardiovascular risks of pediatric hypertension, more emphasis on the need for childhood BP screening and management, new public health initiatives being implemented, and increasing research interest and funding. This article summarizes what is currently known about pediatric hypertension, the existing knowledge-practice gaps, and ongoing research aimed at improving future kidney and cardiovascular health.

Hypertension is one of the most common causes of preventable global disease and death.^1^, ^2^, ^3^ Global hypertension prevalence has doubled from 1990 to 2019, but less than half of the patients with hypertension are diagnosed and less than one-quarter are adequately controlled.^2^ Significant global disparities exist, with lower rates of hypertension diagnosis, treatment, and control in low- and middle-income countries.^2^^,^^4^ There is strong evidence that pediatric hypertension tracks into adulthood and is associated with premature cardiovascular and kidney diseases.^5^, ^6^, ^7^, ^8^, ^9^, ^10^, ^11^, ^12^ Therefore, early detection and adequate management of pediatric hypertension should be prioritized.

### Hypertension Prevalence

The prevalence of pediatric hypertension has increased in recent decades, contributed partly by rising childhood obesity.^13^^,^^14^ However, rates of pediatric hypertension depend on the definition used, which have changed over time and vary globally (Table 1).^5^^,^^15^^,^^16^^,^^17^ Without direct evidence linking specific BP thresholds to cardiovascular outcomes, pediatric hypertension is defined using normative distributions. Between 3% and 5% of children and adolescents have hypertension and 10% and 14% have elevated BP levels (“prehypertension”).^13^^,^^14^^,^^18^, ^19^, ^20^ In a global meta-analysis, the pooled prevalence of hypertension was 4.0% and prehypertension was 9.7%.^14^ Hypertension prevalence increased from 1.3% (1990–1999) to 6.0% (2010–2014).

**Table 1:** Classification of office-based BP in children and adolescents by the American Academy of Pediatrics 2017, European Society of Hypertension 2016, and Hypertension Canada 2020 guidelines

Guidelines	American Academy of Pediatrics (2017)^5^	European Society of Hypertension (2016)^15^	Hypertension Canada (2020)^16^
BP screening and measurement	- Annual BP measurement in children ≥3 yr of age, or at every visit if risk factors for hypertension - Oscillometric methods can be used for screening, but must be confirmed by auscultatory method - Elevated BP should be confirmed on 3 separate clinic visits - ABPM recommended	- BP measurement should be performed in children ≥3 yr of age, can repeat every 2 yr if BP normal - Auscultatory method preferred - Elevated BP should be confirmed on 3 separate clinic visits - ABPM recommended	- BP should be regularly measured in children ≥3 yr of age, no recommendation on screening frequency - Oscillometric methods can be used for screening, but must be confirmed by auscultatory method - Elevated BP should be confirmed on 3 separate clinic visits - ABPM should be considered
Hypertension threshold	≥95th percentile (<13 yr) Or ≥130/80 (≥13 yr)	≥95th percentile (<16 y) Or ≥140/90 mm Hg (≥16 y)	≥95th percentile Or >120/80 mm Hg (6–11 yr) Or >130/85 mm Hg (≥12 yr)
Target BP (general pediatric population)	<90th percentile (<13 yr) Or <130/80 (≥13 yr)	<95th percentile recommended <90th percentile should be considered	<95th percentile <90th percentile (for patients with risk factors or target organ damage)
Target BP (pediatric CKD)	24-h MAP (by ABPM) of <50th percentile	<75th percentile (nonproteinuric CKD) <50th percentile (proteinuric CKD)	<90th percentile

ABPM, ambulatory blood pressure monitoring; BP, blood pressure; CKD, chronic kidney disease; MAP, mean arterial pressure.

### State of Pediatric Hypertension Care

Despite the high prevalence, pediatric hypertension care remains suboptimal (Figure 1). There are conflicting recommendations on pediatric BP screening. Although the most recent guidelines of the American Academy of Pediatrics, European Society of Hypertension, and Hypertension Canada recommend yearly BP screening for healthy children ≥ 3 years old (Table 1),^5^^,^^15^^,^^21^ both the United States Preventative Services Taskforce and the United Kingdom National Screening Committee do not recommend screening.^22^^,^^23^ In theory, a good screening test should be safe, inexpensive, widely available, and able to detect preclinical disease with effective treatment.^2^ All of these characteristics apply to pediatric office-based BP measurement. Pediatric BP screening may also help detect hypertension comorbidities and causes of secondary hypertension. BP screening and follow-up are incomplete. In 2 Canadian studies of 9667 and 378,002 children, respectively, only 15% to 33% of children had annual BP measurement.^19^^,^^24^ Only 5% to 56% of children have appropriate follow-up after elevated BP level measurement.^19^^,^^24^, ^25^, ^26^, ^27^ Less than 25% of children with hypertension are accurately diagnosed, less than half receive lifestyle counseling, and only 6% are prescribed antihypertensive medication.^19^^,^^25^^,^^26^^,^^28^, ^29^, ^30^ Clear challenges and knowledge-practice gaps exist in pediatric hypertension care (Figure 1).

**Figure 1:**
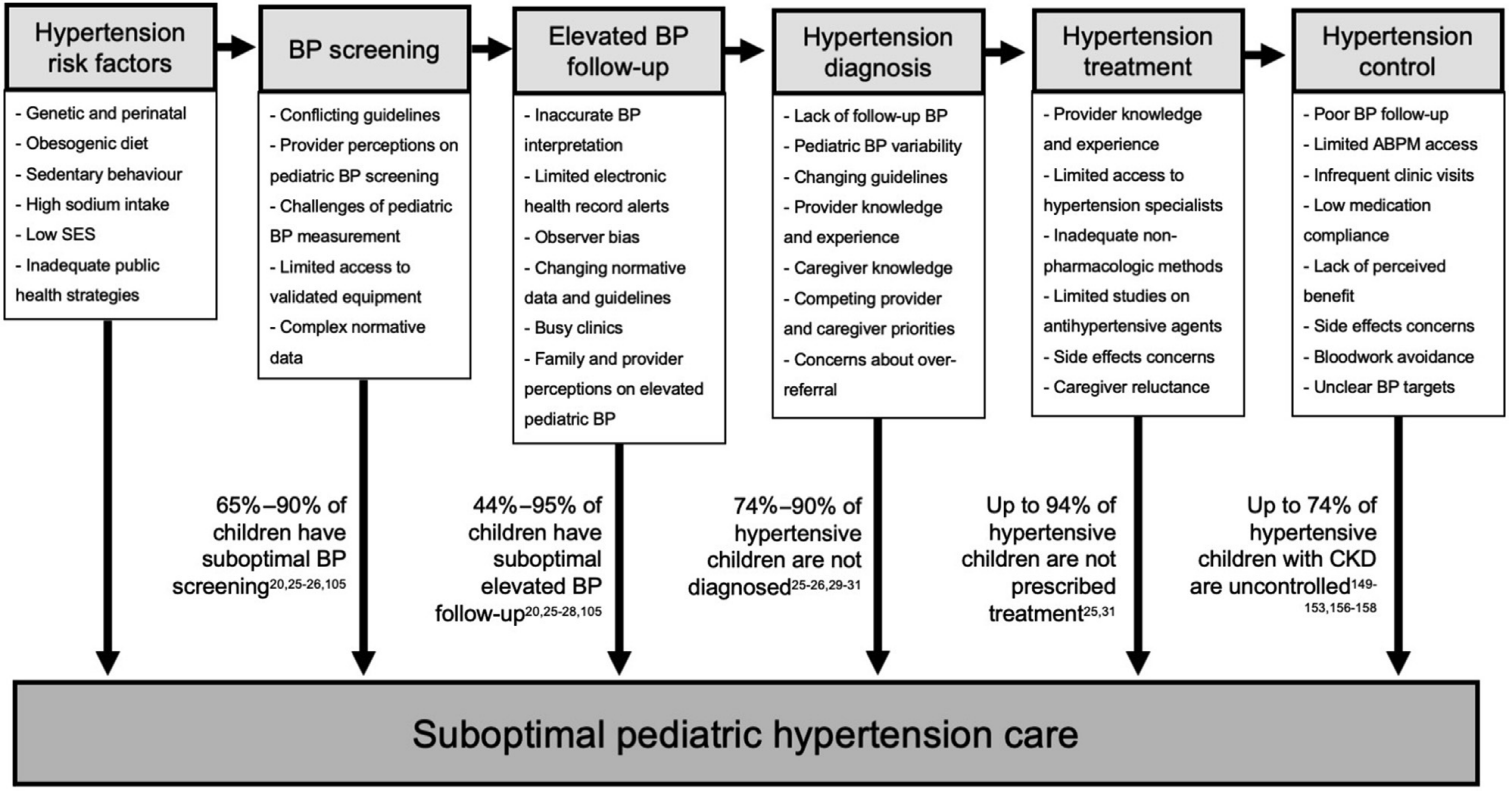
Barriers and knowledge-practice gaps leading to suboptimal pediatric hypertension care. Estimates are provided for the proportion of children in each of the referenced studies that fit the stated description. These are included to provide approximations of the proportion of children who receive suboptimal care at each stage, including population BP screening, follow-up of elevated BP readings, diagnosis of hypertension, management of hypertension, and adequate BP control. Details of the specific populations included and study methods can be found in the references provided. Aside from the studies of hypertension control in children with CKD, all of the other referenced studies were conducted in the United States (and 1 Canadian study^21^). There may be significant global practice variation in pediatric hypertension care. Without data from other countries, it is not possible to extrapolate beyond the North American context. We used existing guidelines at the time of study publication to define what proportion of children received “suboptimal care.” These guidelines were the NHLBI fourth report (from 2004 to 2017), the AAP 2017 guidelines (from 2017 to present), and the KDIGO guidelines (for children with CKD).^5^^,^^17^^,^^22^ ABPM, ambulatory BP monitoring; AAP, American Academy of Pediatrics; BP, blood pressure; CKD, chronic kidney disease; KDIGO, Kidney Disease Improving Global Outcomes; NHLBI, National Heart Lung and Blood Institute.

### Determinants of Pediatric Hypertension

The cause of increasing pediatric hypertension is multifactorial. Primary hypertension accounts for 50% to 90% of cases and is more common in older children and adolescents.^31^, ^32^, ^33^ However, secondary causes should be excluded after hypertension diagnosis, particularly in treatment-resistant and young children (Table 2).

**Table 2:** Causes of pediatric hypertension

Primary (“essential”) hypertension
Risk factors:
▪ Obesity
▪ Sedentary lifestyle
▪ High sodium intake and sodium sensitivity
▪ Low socioeconomic status and food insecurity
▪ Tobacco use
▪ Males
▪ Minority ethnic groups (e.g., Black, Hispanic, and Asian children)
▪ Family history of hypertension
▪ Perinatal factors (e.g., low birthweight, prematurity, maternal BP, and age)

**Renal disease**

▪ Acute kidney injury
▪ Chronic kidney disease
▪ Renal scarring (e.g., previous pyelonephritis, trauma)
▪ Glomerulonephritis
▪ Renal vasculitis
▪ Nephrotic syndrome
▪ Polycystic kidney disease
▪ CAKUT
▪ Hemolytic-uremic syndrome

**Endocrine disease**

▪ Congenital adrenal hyperplasia
▪ Cushing syndrome
▪ Familial hyperaldosteronism
▪ Apparent mineralocorticoid excess
▪ Liddle, Geller, and Gordon syndromes
▪ Hyperthyroidism and hypothyroidism
▪ Hyperparathyroidism
▪ Diabetes mellitus

**Vascular disease**

▪ Aortic coarctation
▪ Renal artery stenosis
▪ Renal vein thrombosis
▪ Midaortic syndrome
▪ Other genetic/syndromic conditions (e.g., neurofibromatosis, tuberous sclerosis, Williams, Turner, Alagille)

**Oncologic disease**

▪ Wilms tumor
▪ Phaeochromocytoma, paraganglioma
▪ Neuroblastoma
▪ Reninoma

**Neurologic disease**

▪ Raised intracranial pressure
▪ Autonomic system dysfunction (e.g., Guillain-Barré syndrome)

**Medications and toxins**

▪ Iatrogenic volume and sodium loading (e.g., excess 0.9% saline administration)
▪ Corticosteroids
▪ Stimulants
▪ Sympathomimetics
▪ Oral contraceptives
▪ Nicotine
▪ Cocaine
▪ Caffeine
▪ Licorice
▪ Heavy metal toxicity (e.g., lead, cadmium, mercury)

**Other causes**

▪ Obstructive sleep apnea
▪ Pain, anxiety

BP, blood pressure; CAKUT, congenital anomalies of the kidneys and urinary tract.

#### Genetic and Perinatal Factors

Familial aggregation of hypertension is well known. Genetic factors significantly contribute, as demonstrated by the stronger association between parent/child BP than between spouses^34^ and lack of BP correlation between parents and adopted children.^35^^,^^36^ Familial and twin studies demonstrate that BP heritability is ∼30% to 50%.^37^, ^38^, ^39^, ^40^ Genome-wide association studies have identified many BP loci, although each individually accounts for small BP differences (<1 mm Hg).^41^, ^42^, ^43^ Epigenetic and gene-environment interactions are likely significant. Multiple perinatal factors are associated with childhood BP, including low birthweight, prematurity, and maternal factors (i.e., pre-eclampsia, BP, age, and body mass index).^44^, ^45^, ^46^, ^47^ These factors can impair nephrogenesis, predisposing affected individuals to hypertension and salt sensitivity.^48^, ^49^, ^50^, ^51^, ^52^ A systematic review by Rabe *et al.*^53^ found some evidence that maternal age, BP, body mass index, and smoking were associated with neonatal BP, although there are insufficient published data. Postnatally, breastfeeding has been consistently associated with lower childhood BP level.^53^, ^54^, ^55^, ^56^, ^57^, ^58^ Lower sodium exposure in breastfed infants is a potential contributor.^59^

#### Obesity, Diet, and Physical Activity

Obesity is a major risk factor for pediatric hypertension.^60^, ^61^, ^62^, ^63^, ^64^ The risk of hypertension is 2.6× greater in overweight children (body mass index-for-age ≥85th percentile)^65^ and 9.2× greater in obese children (≥95th percentile).^65^ Obesity-hypertension begins early in children (<5 years).^66^, ^67^, ^68^ The mechanisms of obesity-hypertension are complex but include impaired sodium handling, sympathetic nervous system overactivation, oxidative stress, hemodynamic changes, and renal/endocrine dysfunction.^69^ Physical activity is inversely associated with childhood obesity and directly counteracts obesity-hypertension mechanisms.^70^

Dietary sodium intake is also closely associated with BP.^13^^,^^71^^,^^72^ In North American children, daily sodium intake is ∼3000 mg to 3400 mg (approximately 2–3 times the recommended daily intake), and three-quarters of Canadian children exceed upper tolerable limits.^73^, ^74^, ^75^ Approximately 80% of dietary sodium comes from packaged and processed foods.^74^ Salt sensitivity (i.e., BP rise following sodium intake) is an important mediator.^76^ Individuals with hypertension, obesity, low birthweight, and African Americans have greater salt sensitivity, which is associated with increased target organ damage, cardiovascular disease, and mortality.^77^, ^78^, ^79^, ^80^ However, assessing an individual’s salt sensitivity is still clinically impractical. Salt sensitivity testing requires either strict adherence to high and low sodium diets on a prolonged outpatient protocol, or i.v. sodium loading studies, neither of which are practical for most children with hypertension.

#### Ethnic Differences, Socioeconomic Status, and the Developing World

Significant racial and ethnic BP differences are well characterized in adults.^81^, ^82^, ^83^, ^84^ Minority ethnic groups are consistently shown to have more hypertension and worse BP control.^81^, ^82^, ^83^^,^^85^^,^^86^ BP level is also higher among Black, Hispanic, and Asian children.^87^, ^88^, ^89^ Associations are reported between low socioeconomic status, parental income, and education with childhood BP.^90^, ^91^, ^92^, ^93^, ^94^ Kelly *et al.*^94^ found that socioeconomic status improvement into adulthood significantly decreased BP level. In a meta-analysis by Beltrán *et al.*,^95^ food insecurity was significantly associated with hypertension (odds ratio 1.44, 95% CI 1.16–1.79). Ethnic and socioeconomic differences may relate to diet (i.e., access to high-quality foods and salt intake), obesity, prenatal factors, timing of sexual maturity, psychological stress, and health care access.

Although hypertension detection and management have improved significantly in high-income countries, the same is not true in the developing world.^2^^,^^4^ High rates of tobacco use, salt intake, and obesity contribute to high hypertension prevalence, whereas low health literacy and limited health care access are major barriers to adequate hypertension control. These issues may be further exacerbated by rapid urbanization in low- and middle-income countries.^4^^,^^96^, ^97^, ^98^

### Pediatric Hypertension Outcomes

Although uncontrolled adult hypertension is clearly associated with cardiovascular disease and mortality, there is limited direct evidence for hard cardiovascular outcomes in pediatric hypertension. Demonstrating this association would require a large cohort of children with hypertension, many decades of follow-up, and high participant retention, which is neither financially nor practically feasible. However, there is substantial evidence that pediatric BP tracks into adulthood and that pediatric hypertension increases the risk of subclinical cardiovascular disease (“target organ damage”).^5^, ^6^, ^7^^,^^99^

#### BP Tracking

Children with hypertension and adolescents are more likely to become adults with hypertension, although the correlation is incomplete.^9^, ^10^, ^11^, ^12^^,^^95^^,^^100^, ^101^, ^102^, ^103^, ^104^ Reported correlation coefficients between childhood and adult BP are 0.2 to 0.5 (weak-to-moderate correlation).^9^^,^^10^^,^^12^^,^^94^^,^^104^ However, BP tracking between adolescence and adulthood and among obese individuals is stronger.^10^, ^11^, ^12^ Individuals with persistent hypertension (through childhood and adolescence) have a 7.6× greater odds of adult hypertension.^12^ Of note, many children with hypertension have BP normalization over time. In 1881 children with hypertension, nearly three-quarters had normal or only elevated BP level in the following 3 years.^105^ Factors associated with BP normalization include decreased body mass index, increased vegetable intake, decreased alcohol use, and improved socioeconomic status.^95^

#### Subclinical Cardiovascular Outcomes

Pediatric hypertension is associated with target organ damage, which in turn is associated with future cardiovascular disease. Children with hypertension have higher left ventricular mass index and left ventricular hypertrophy (LVH).^106^, ^107^, ^108^, ^109^, ^110^, ^111^, ^112^, ^113^, ^114^ Between 5% and 50% of children with hypertension have LVH, and a dose-dependent relationship is shown with increasing BP severity.^115^^,^^116^ Children with hypertension also have increased carotid intima-media thickness,^117^, ^118^, ^119^, ^120^ higher pulse-wave velocity,^121^^,^^122^ arterial calcification and atherosclerotic changes,^123^, ^124^, ^125^, ^126^, ^127^ retinal microvascular disease,^128^, ^129^, ^130^ and microalbuminuria.^131^^,^^132^ In a large cohort of Israeli military recruits (16–19 years old), adolescent hypertension was associated with an increased risk of long-term kidney failure, as defined by dialysis and transplant registries (adjusted hazard ratio 1.98, 95% CI 1.42–2.77), although the absolute risk was low (0.5%).^8^ In a meta-analysis of 19 studies, Yang *et al.*^6^ found that elevated office BP level in children was significantly associated with adult LVH, carotid intima-media thickness, and pulse-wave velocity, as well as cardiovascular events and mortality. In another meta-analysis by Chung *et al.*,^99^ children with ambulatory hypertension (defined by ambulatory BP monitoring [ABPM]) had significantly increased carotid intima-media thickness, pulse-wave velocity, left ventricular mass index, and LVH rates. Overall, there is strong evidence that pediatric hypertension is associated with adverse subclinical cardiovascular outcomes. In adults, these subclinical cardiovascular outcomes are consistently associated with an increased risk of cardiovascular events.^127^^,^^133^, ^134^, ^135^, ^136^ However, pediatric data demonstrating a direct association between these subclinical outcomes, mortality, and clinical cardiovascular events are lacking. To further explore these associations, the Study of High Blood Pressure in Pediatrics: Adult Hypertension Onset in Youth study is establishing a multiethnic cohort of adolescents to define optimal BP thresholds and evaluate markers of hypertensive target organ damage.^137^ Fortunately, antihypertensive treatment is shown to improve LVH in pediatric studies, including patients with CKD.^138^, ^139^, ^140^, ^141^, ^142^

#### Hypertension in Pediatric CKD

Hypertension is strongly associated with CKD progression in children and adults, and BP lowering prevents CKD progression.^143^, ^144^, ^145^, ^146^, ^147^, ^148^ In childhood CKD, hypertension is common (48%–70%),^149^, ^150^, ^151^, ^152^, ^153^, ^154^, ^155^ and <50% are adequately controlled.^149^, ^150^, ^151^, ^152^, ^153^^,^^156^, ^157^, ^158^ In the Chronic Kidney Disease in Children study, 83% of the participants had ambulatory hypertension (including abnormal BP load) and 35% had masked hypertension.^157^ The optimal BP target in pediatric CKD has not been established (Table 1). The Kidney Disease: Improving Global Outcomes 2021 guidelines recommend a systolic BP target <120 mm Hg for adults with hypertension and CKD.^159^ In children, the Kidney Disease: Improving Global Outcomes guidelines recommend targeting a 24-hour mean arterial pressure (MAP) <50th percentile (level 2C; weak recommendation, low-quality evidence). This is supported by the Effect of Strict Blood Pressure Control and ACE Inhibition on the Progression of CKD in Pediatric Patients (ESCAPE) trial (385 participants), which demonstrated lower CKD progression with intensive BP control, particularly in proteinuric kidney disease.^144^ Recent data from the Chronic Kidney Disease in Children study also found that high MAP (>90th percentile) was associated with CKD progression.^149^ However, using ABPM-based targets for pediatric hypertension management is impractical and limits global applicability. The 2016 guidelines of the European Society of Hypertension instead recommend an office-based BP target of ≤75th percentile (nonproteinuric CKD) and of ≤50th percentile (proteinuric CKD).^15^

### Challenges in BP Measurement and Interpretation

Standardized, reliable BP measurement is critical to hypertension diagnosis. Unfortunately, pediatric BP measurement is challenging. In North America, pediatric BP screening, elevated BP level follow-up, and hypertension diagnosis are suboptimal (Figure 1). There are minimal data on the extent of pediatric hypertension screening in low- to middle-income countries, where underdiagnosis may be more prevalent.

#### Office-Based BP Measurement

Office-based BP was traditionally measured using mercury sphygmomanometers. These have been gradually replaced by aneroid sphygmomanometers, although there are limited pediatric validation data, and these require routine calibration. Oscillometric devices are popular, given their ease of use and consistency. They overcome observer bias and prevent terminal digit preference (i.e., rounding measurements to certain digits). However, oscillometric devices estimate systolic and diastolic BP levels using proprietary formulas by measuring MAP and pulse pressure. Significant differences may exist between oscillometric devices, and they tend to overestimate pediatric BP level by 3 to 10 mm Hg.^160^, ^161^, ^162^ Normative BP data are typically derived by auscultatory methods, so abnormal oscillometric BP should be confirmed by auscultation.^5^^,^^15^^,^^16^ Repeated or averaged BP measurements are also more reliable, because BP level can decrease during a single visit.^163^^,^^164^ Newer automated devices can repeat BP measurements in clinic without an observer present and have been shown to reduce white coat phenomenon in adults.^165^ Elevated BP level should be confirmed on 3 separate visits to diagnose hypertension.^88^^,^^106^ Interpretation of pediatric BP is also challenging; with large reference tables, changing normative data, and conflicting definitions of pediatric hypertension. The development of accessible tools, including simple BP screening tables and mobile applications (e.g., PedBP), has simplified diagnosis.^5^ Although convenient, office-based BP provides only a snapshot of a patient’s BP. In the Study of High Blood Pressure in Pediatrics: Adult Hypertension Onset in Youth, office-based BP level ≥85th percentile was most predictive of ambulatory hypertension and may be considered as a threshold for ABPM.^166^

#### Home and ABPM

Ambulatory BP is more reproducible and better associated with target organ damage than office-based BP.^167^, ^168^, ^169^ There are limited pediatric data on home BP monitoring (HBPM),^170^ although it is commonly used (>70% of surveyed German pediatric nephrologists^171^) and has gained popularity during the COVID-19 pandemic.^172^ HBPM provides a more longitudinal BP assessment than either office-based BP or ABPM, is cost-effective, and is well tolerated.^173^^,^^174^ Although not recommended for pediatric hypertension diagnosis, HBPM can help detect white coat or masked hypertension. HBPM is also useful for BP monitoring in patients with hypertension, especially when strict BP control is desired.^5^^,^^15^ However, there can be reporting bias, the optimal timing of measurements is unknown, and validated pediatric devices and cuff sizes are lacking.^15^ HBPM should be supported by adequate caregiver training and device calibration with office-based auscultatory BP. Telemedicine strategies for hypertension management, including BP telemonitoring, have been shown to be feasible and associated with improved BP control in adults with hypertension.^175^ However, there are minimal data on the use of BP telemonitoring in children. Incorporation of these strategies could improve access to pediatric hypertension care, may promote disease self-management, and enhance lifestyle modification. However, these potential benefits are balanced against limited access to validated pediatric home BP devices, a lack of standardized protocols for HBPM, few pediatric telemedicine services, regulatory and privacy issues, and provider reimbursement considerations.^175^ Further research on the clinical application of HBPM in children is needed to facilitate BP telemonitoring programs.^170^

ABPM is the gold standard for adults and is recommended by the European Society of Hypertension and the American Academy of Pediatrics for children (>5 years).^5^^,^^15^ ABPM is well correlated with target organ damage^167^, ^168^, ^169^ and is reliable in pediatric CKD.^144^ It can detect nocturnal and masked hypertension, which are both more common in CKD.^158^^,^^159^ An “ABPM-first” approach for pediatric hypertension referrals (i.e., performing ABPM in all new referrals to confirm hypertension before consultation and to avoid unnecessary expensive secondary hypertension workups) is a potential cost-saving strategy.^176^ However, there are limitations to widespread pediatric ABPM utilization. There are few validated pediatric devices, costs are prohibitive, and global access is limited. Existing ABPM normative data are also problematic. Current normative data were derived from a relatively small Caucasian German population.^177^^,^^178^ There are minimal data for children <120 cm in height, and concerns exist regarding low diastolic BP variation in this cohort. BP varies by ethnicity and geographic region. Xi *et al.*^179^ attempted to create international normative BP data from 52,636 children in 7 countries using office-based BP methods. Median systolic/diastolic BP levels varied up to 10 mm Hg between countries, with India and Poland having the highest BP level. Based on these differences in BP by ethnicity, existing ABPM normative data may not be applicable to non-Caucasian children. Yip *et al.*^180^ developed ABPM normative data for East Asian children in Hong Kong, and BP values were 5 to 6 mm Hg higher than those in Caucasian children. There are ongoing efforts to develop validated normative data sets in other ethnicities, including South Asian children in Canada in the Ambulatory blood pressure monitoring for SoutH Asian children study.^181^

Another limitation is the existing pediatric ABPM classification. In adults, ambulatory hypertension is defined by simple thresholds (i.e., mean wake BP > 130/80 mm Hg, sleep BP > 110/65, or 24-hour BP > 125/75), that predict cardiovascular events.^182^^,^^183^ In the pediatric American Heart Association guidelines, ambulatory hypertension is categorized by mean BP and BP load.^169^ However, up to 20% to 40% of children are unclassified using these criteria and hypertension thresholds may be higher than adult thresholds for children ≥12 years old.^184^, ^185^, ^186^ There is emerging evidence that isolated elevated BP load is not significantly associated with target organ damage.^185^^,^^187^^,^^188^ Removing BP load criteria and using adult thresholds for adolescents would simplify ABPM interpretation.^186^^,^^188^ Because oscillometric ABPM devices measure MAP, it may also be preferable to classify ABPM using MAP, instead of calculated systolic/diastolic BP.

### Pediatric Hypertension Management

Optimal pediatric BP thresholds are unknown (Table 1), but the goal is to reduce BP to a level that minimizes cardiovascular and kidney disease risks. In adults with hypertension, the Systolic Blood Pressure Intervention Trial demonstrated that intensive BP control (systolic BP target <120 mm Hg) was associated with a significantly lower risk of cardiovascular outcomes,^189^ which has led to the incorporation of lower BP targets in recent adult hypertension guidelines.^182^^,^^190^ Strategies to improve pediatric hypertension typically address the individual level. However, pediatric hypertension is a growing pandemic, and effective population-based interventions are essential to address the global disease burden (Figure 2). Improving awareness of pediatric hypertension among primary care physicians, community organizations, and families may increase detection, provide earlier treatment opportunities, and mitigate adverse consequences.

**Figure 2:**
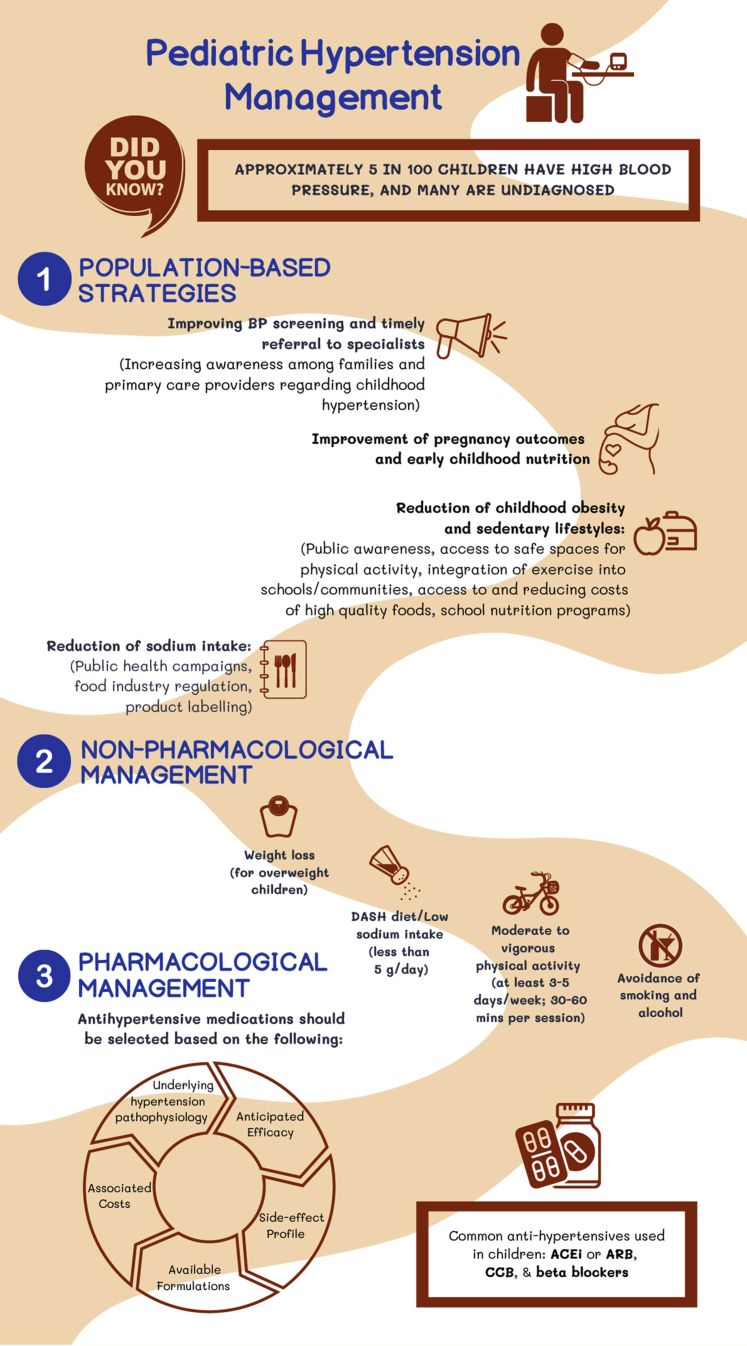
Strategies to improve global pediatric hypertension care. ACEi, angiotensin-converting enzyme inhibitor; ARB, angiotensin receptor blocker; CCB, calcium channel blocker.

#### Population-Based Strategies

Population-based sodium reduction strategies are highly cost-effective.^191^ In Finland and the United Kingdom, public health campaigns, food industry regulations, and product labeling have successfully decreased population sodium intake by 15% to 40%.^192^ Public health strategies should also address childhood obesity and sedentary lifestyles, including awareness campaigns, creating safe spaces for physical activity, integrating exercise into schools and communities, and improving access to high-quality nutrition (e.g., through food taxes, subsidies, and school-based programs).^4^ In 2013, the World Health Organization created a Global Action Plan for the control of noncommunicable diseases and described a series of “best buy” interventions, considered to be the most cost effective and feasible. These include reducing tobacco and alcohol use, reducing salt and transfat intake, and public health physical activity campaigns.^193^ In rural South Asia, the COBRA-BPS trial found that a multicomponent community hypertension intervention (including community health education, BP monitoring, provider training in hypertension management, designated hypertension clinics, and additional funding) significantly improved hypertension control and was cost effective.^194^^,^^195^ Strategies to improve pregnancy outcomes, early childhood education, and nutrition are also critical. The Carolina Abecedarian Project found that an early childhood education and nutrition program significantly decreased adult hypertension.^196^

#### Nonpharmacologic Management

Effective nonpharmacologic strategies for pediatric BP lowering include weight loss (for overweight children), regular physical activity, reduced sodium intake, the Dietary Approaches to Stop Hypertension diet, and smoking/alcohol avoidance (Figure 2). There is strong evidence in both adults and children that dietary sodium reduction is associated with improved BP control, in a dose-dependent relationship.^197^, ^198^, ^199^ In 2 pediatric meta-analyses (966 and 58,531 patients respectively), reduced dietary sodium intake was associated with small, but significant BP reductions (∼1 mm Hg).^199^^,^^200^ The association between BP and sodium intake was stronger in overweight children and children with low potassium intake.^199^ Achieving sustainable sodium reductions is challenging, given the sodium content in processed foods.^75^ Although optimal sodium reduction targets for children are uncertain, the National Academic of Sciences, Engineering and Medicine have recommended Chronic Disease Risk Reduction Intake limits, based on extrapolated adult data (1–3 years: <1200 mg/d; 4–8 years: <1500 mg/d; 9–13 years: <1800 mg/d; 14–18 years: <2300 mg/d).^76^ A practical approach for sodium reduction is to recommend a no added salt diet, a reduction of high-salt, processed foods, and to provide education to families regarding food label interpretation. Self-reported sodium intake is also inaccurate.^201^ Urine sodium excretion is more reliable, and novel formulas to estimate sodium excretion from spot urine samples are available.^201^, ^202^, ^203^ Higher sodium excretion is associated with major cardiovascular events.^204^

The Dietary Approaches to Stop Hypertension diet was designed in the 1990s as an optimal BP-lowering diet for adults.^205^ The Dietary Approaches to Stop Hypertension diet promotes consumption of vegetables, fruit, lean meat, and dairy, and reduces intake of sodium, saturated fat, added sugars, and highly processed foods. The Dietary Approaches to Stop Hypertension diet has also been shown to improve BP in children and adolescents, although there are limited published data.^206^^,^^207^ Regular physical activity has also been shown to reduce BP in children and adolescents with hypertension.^208^, ^209^, ^210^ However, the results of published studies are inconsistent and the effect size is generally small. Physical activity interventions appear to be more effective when combined with diet or weight loss programs.^209^

#### Pharmacologic Management

Nonpharmacologic interventions should be optimized before antihypertensive treatment. Antihypertensive medications should be selected based on underlying hypertension pathophysiology, anticipated efficacy, side effects, available formulations, and associated costs.^5^ Long-acting medications and simplified dosing schedules can improve compliance. Few pediatric trials compare antihypertensive medications. A systematic review by Simonetti *et al.*^211^ found that angiotensin-converting enzyme inhibitors, angiotensin II receptor blockers, and calcium channel blockers had similar antihypertensive efficacy. A 2014 Cochrane review found that angiotensin-converting enzyme inhibitors, angiotensin II receptor blockers, and beta-blockers each significantly reduced BP versus placebo, whereas calcium channel blockers did not.^212^ A subsequent network meta-analysis by Burrello *et al.*^213^ found similar BP reductions across antihypertensive classes, but only renin-angiotensin-aldosterone system inhibitors significantly reduced BP versus placebo. Generally, renin-angiotensin-aldosterone system inhibitors are considered first-line pediatric antihypertensives, particularly in CKD.^144^ Calcium channel blockers are considered for sexually active adolescent females or if laboratory surveillance (for renin-angiotensin-aldosterone system inhibitors) is poorly tolerated.^5^^,^^15^^,^^16^ Samuel *et al.*^214^ described a novel antihypertensive selection approach, by conducting serial n-of-1 trials in 42 children with ABPM, identifying each patient’s “preferred” medication (49% lisinopril, 24% amlodipine, and 12% hydrochlorothiazide).

### Future Directions and Knowledge Gaps

Despite significant advances in pediatric hypertension research, knowledge gaps persist. It is unclear what BP thresholds are associated with cardiovascular outcomes and should be targeted. We must determine the optimal intermediate markers (e.g., LVH) for predicting cardiovascular events. Because BP tracking and target organ damage are incomplete, we must identify relevant predictive factors. We should also evaluate the effect of hypertension duration on cardiovascular outcomes. It remains unclear if transient hypertension (i.e., during childhood chemotherapy) has long-term risks and warrants treatment. The optimal role and timing of HBPM and ABPM are unclear, and progress is needed to validate and improve access to pediatric devices. Additional ABPM normative data sets are needed, in diverse ethnic populations. Finally, further trials are needed to evaluate various antihypertensive medications, particularly among specific populations (e.g., obesity, nonproteinuric CKD, and congenital heart disease). Fortunately, ongoing research will help answer some of these questions, including the Study of High Blood Pressure in Pediatrics: Adult Hypertension Onset in Youth study,^138^ further Chronic Kidney Disease in Children analyses (https://statepi.jhsph.edu/ckid), the Ambulatory blood pressure monitoring for SoutH Asian children study, the Pediatric Hypertension Registry, prospective longitudinal cohorts including the Young Finns study (https://youngfinnsstudy.utu.fi), and novel intervention trials (e.g., pharmacist- or youth-led programs, n-of-1 medication trials, and clinical decision support tools).

## Disclosure

All the authors declared no competing interests.
